# Chromosome-scale genome assembly of the bed bug *Cimex lectularius* sheds light on a key insecticide resistance locus

**DOI:** 10.1093/g3journal/jkaf161

**Published:** 2025-07-18

**Authors:** Chloé Haberkorn, Julien Varaldi, Oriane Plantec, Nelly Burlet, Ines Amdouni, Elsa Baligand, Albert Ndour, Louis Sanglier, Christine Oger-Desfeux, Fabrice Vavre

**Affiliations:** Universite Claude Bernard Lyon 1, LBBE, UMR 5558, CNRS, VetAgro Sup, Villeurbanne 69622, France; Department of Zoology, Stockholm University, Svante Arrheniusväg 18 B, Stockholm 106 91, Sweden; Universite Claude Bernard Lyon 1, LBBE, UMR 5558, CNRS, VetAgro Sup, Villeurbanne 69622, France; INSA Lyon, Master internship, Villeurbanne 69621, France; Universite Claude Bernard Lyon 1, LBBE, UMR 5558, CNRS, VetAgro Sup, Villeurbanne 69622, France; Master bioinfo@lyon, Universite Claude Bernard Lyon 1, Villeurbanne 69622, France; Master bioinfo@lyon, Universite Claude Bernard Lyon 1, Villeurbanne 69622, France; Master bioinfo@lyon, Universite Claude Bernard Lyon 1, Villeurbanne 69622, France; Master bioinfo@lyon, Universite Claude Bernard Lyon 1, Villeurbanne 69622, France; Universite Claude Bernard Lyon 1, PRABI-AMSB, FR3728 BIOEENVIS, Villeurbanne 69622, France; Universite Claude Bernard Lyon 1, LBBE, UMR 5558, CNRS, VetAgro Sup, Villeurbanne 69622, France

**Keywords:** bed bug, genome assembly, chromosome-scale assembly, insecticide, resistance, Hi-C, Cimex lectularius

## Abstract

The population densities of the common bed bug, *Cimex lectularius*, have recently exploded worldwide. This demographic boom is mostly due to the evolution of insecticide resistance, which appears to be mainly driven by one autosomal locus in this species, identified by a Quantitative Trait Loci analysis. However, the exact gene content of this locus is still unclear, in particular regarding the inclusion of the voltage-gated sodium channel gene, due to uncertainty in previous assemblies available. To resolve this ambiguity, and more generally to provide useful resources to fight this hematophagous human parasite, we combined short, long, and Hi-C reads to produce a chromosome-scale assembly for this species. Three competing assembly strategies were used, all of which resulted in 13 autosomes plus two X chromosomes, consistent with previous cytological studies and a very recent chromosome-scale assembly. The best assembly had a total length of 507 Mb, an N50 of 35 Mb, encoded 98% of complete BUSCO genes, and covered 99% of the previous reference genome. This chromosome-scale assembly revealed that the main insecticide-resistance locus does indeed contain the voltage-gated sodium channel gene, as well as other genes possibly involved in insecticide resistance. Additionally, a population genomics analysis showed that this 7.65 Mb locus is highly differentiated between insecticide-resistant and susceptible strains, confirming previous results. We hope this high-quality, complete, and annotated genome of *C. lectularius* will serve as a useful resource to understand the mechanisms of insecticide resistance evolution and, more generally, better control bed bug populations.

## Introduction

The population densities of *Cimex lectularius* associated with humans have recently exploded worldwide. This is most likely due to several factors, including the increase in traveling or in second-hand markets. However, among these factors, the evolution of resistance to insecticide seems to play a major role (Dang et al. 2017). Thus, identifying the genetic determinants of this resistance is of crucial importance in order to efficiently control bed bug populations. To reach this goal, genomic resources constitute a necessary tool.

Since 2016, a 510 Mb reference genome is available for *C. lectularius* (Harlan strain) (Fountain et al. 2016). However, this assembly is relatively fragmented (1,462 scaffolds, L50 = 100). Because of this limitation, a linkage map for this assembly using 334 RAD markers was constructed (Fountain et al. 2016), allowing them to group the scaffolds into 14 linkage groups, putatively corresponding to 14 chromosomes, spanning 65% of the first version of the genome (Clec_1.0). In addition, based on a Quantitative Trait Loci (QTL) mapping approach relying on the same genetic markers analyzed in an F2 cross involving insecticide-resistant and insecticide-susceptible strains, the same authors identified a major QTL on LG12. This large QTL (11.1 cM corresponding roughly to 10 Mb) encoded various genes known to be involved in insecticide resistance in insects, including the well-known voltage-gated sodium channel (VGSC). This gene encodes the protein targeted by several insecticides (including pyrethroids) and several mutations of this gene confer resistance to insecticide (phenotype known as knock-down resistance, *kdr*) in various insect models (Dong et al. 2014), including bed bugs (Dang et al. 2017). This locus is thus one of the major candidate responsible for the evolution of insecticide resistance in insects in general, and most likely in bedbugs too, although other genes involved in the resistance to other insecticides or involving other mechanisms (detoxification, cuticle thickening) may also play a role (Dang et al. 2017). Nevertheless, due to the rather low density of markers and low sample size analyzed in Fountain et al. (2016), it was unclear which of the gene(s) is(are) really responsible for the resistance phenotype in *C. lectularius*.

In a recent paper, we performed a genome scan comparing two closely related strains (London “Lab” and London “Field”) differing in their resistance phenotype (Haberkorn et al. 2023). Our analysis revealed the presence of a highly differentiated 6 Mb locus, roughly corresponding to the locus identified by Fountain et al. (2016), based on gene content profiles. This region was enriched for genes known to be involved in insecticide resistance, such as the detoxifying enzymes GSTs and P450s, further supporting the hypothesis that this genomic region is involved in insecticide resistance. However, this region did not contain the VGSC gene. To run this previous analysis, we used the most recent and supposedly better reference genome assembly Clec_2.1, combined with the exact same 334 RAD markers used by Fountain et al. (2016) to build a genetic map. This new linkage map also identified 14 LG but the scaffold encoding the *VGSC* gene (NW_019392726) was left unanchored in any putative chromosome. This was due to the fact that the scaffold encoding VGSC in Clec_1.0 (used by Fountain et al. 2016) had been split into pieces in Clec_2.1 assembly, which led to the removal of the RAD markers in the contig. In summary, although our analysis showed that the 1 Mb scaffold encoding *VGSC* was highly differentiated between the resistant and susceptible strains, whether it belonged to the 6 Mb super locus involved in insecticide resistance remained an open question (Haberkorn et al. 2023).

To precisely know where this important insecticide-resistance locus is in the bed bug genome, and more generally to provide a useful resource for the community, we decided to build a chromosome-scale assembly for *C. lectularius* by combining short-reads, long-reads and Hi-C data obtained on the London Lab (LL) “susceptible” strain. This procedure led to a final 507 Mb assembly where 90% of the bases are included in 13 autosomes and two X chromosomes.

During the preparation of this manuscript, chromosome-level assemblies of two haplotypes for the insecticide-susceptible *C. lectularius* Harlan strain were published (Miles et al. 2024). Both haplotypes are composed of 13 autosomes and two X chromosomes, similarly to our assembly. However, because these Harlan haplotype assemblies were not annotated, the question of the position of the insecticide resistance genes was still open. We thus included these additional chromosome-level haplotype assemblies in our analysis to determine the position of the insecticide resistance. An overall comparison of the structure of the three assemblies (1 haplotype from LL, resolved in the present paper + the 2 haplotypes from the Harlan strain) is provided. Finally, we re-used the polymorphism (pool-seq) datasets generated from the pyrethroid insecticide-“resistant” and “susceptible” strains collected in London, to scan the genome of the LL strain for peaks of genetic differentiation.

## Materials and methods

### Strain and sample collection

For all sequencing experiments, we used individuals from the strain LL provided by CimexStore Ltd (Chepstow, UK). This strain was collected in London more than 40 years ago, has been kept in laboratories since, and is susceptible to pyrethroid insecticides according to CimexStore’s statement. This phenotypic status was confirmed by previous bioassays (Haberkorn et al. 2023). Fresh adult females were used for the various DNA extractions.

### Sample preparation and sequencing

#### Illumina sequencing

The DNA of a single female individual was extracted using the Macherey-Nagel kit. The library preparation and Illumina sequencing were performed by MACROGEN (TruSeq Nano DNA kit, 2 × 150 bp). The reads were cleaned using Trimmomatic (parameters: ILLUMINACLIP:adapter.fa:2:30:10:2:keepBothReads) and the final dataset contained 62.4 Gb corresponding to an approximate 125× coverage.

#### MinION sequencing

Due to concentration issues, the sample used for the MinION sequencing was obtained after pooling three individuals. The extraction was performed using the Blood and Cell Culture DNA minikit (Qiagen) following manufacturer’s instructions and the sequencing was performed at the DTAMB (Lyon, France) with the Oxford Nanopore technology (MinION) using the Ligation Sequencing Kit (SQK-LSK109).

The 3,545,571 reads obtained, with a median read length of 1.08 kb, mean of 2.47 kb and N50 of 5.4 kb, were cleaned using Porechop v0.2.4 and chopper (q>10 and length > 700 bp, De Coster and Rademakers 2023) before being further processed. This led to 2,262,935 reads left (700 to 528,839 bp long, total 8.2 Gb) corresponding to an approximate 16× coverage.

#### Hi-C sequencing

A single female was used for the construction of the Hi-C library. The sample used was flash frozen using liquid nitrogen and sent to Arima Genomics company (California, USA) for library preparation and sequencing. The four restriction enzymes sites used were GATC, GANTC, TTAA et CTNAG. 96 Gb (2 × 150 bp) were obtained, resulting in 88.6 Gb after cleaning using Trimmomatic v0.39 (parameters: HEADCROP:5 ILLUMINACLIP: adapter.fa:2:30:10:2:keepBothReads; Bolger et al. 2014).

### Assembly strategy

Three primary assemblies were obtained starting from the same dataset: (i) the long reads were assembled using the software flye (version 2.9.3, Kolmogorov et al. 2019) and the contigs obtained were corrected using pilon (v1.24, Walker et al. 2014) by adding the short Illumina reads, previously mapped using minimap2 (v2.24, Li 2018), (ii) the long reads and the short Illumina reads were assembled using the hybrid assembler WenganM (v0.2, Di Genova et al. 2021), (iii) the long reads and the short Illumina reads were assembled using the hybrid assembler WenganA (v0.2, Di Genova et al. 2021).

Following this primary assembly, haplotypic duplications were removed using purge_dups (v1.2.5, Guan et al. 2020) and each dataset was then scaffolded using the Hi-C data following Arima genomics (https://github.com/ArimaGenomics/mapping_pipeline) and YaHS pipeline (v1.2, Zhou et al. 2023). After visual control of the Hi-C contact map in Juicebox (v2.17, Durand et al. 2016), a few evident incorrect decisions made by the algorithm were corrected by hand for each assembly. This led to three final candidate chromosome-scale assemblies. All three were composed of 15 large chromosome-scale super-scaffolds ranging from 16.4 to 45.2 Mb.

### Annotation

The annotation of the reference genome was transferred to the assemblies using the software liftoff (v1.6.3) using default parameters.

### Quality assessment

The quality of the three genome assemblies was assessed by computing summary statistics using QUAST v5.2.0 (Gurevich et al. 2013) and by running the pipeline BUSCO v5.5.0 (Manni et al. 2021) using the Hemiptera dataset (2,510 genes). These statistics, combined with the result of the annotation, were used to identify the best assembly. The following steps were only performed on this assembly.

### Cleaning the final assembly

The 2,148 unplaced scaffolds (not integrated in any of the 15 chromosomes) were uploaded to the Galaxy web platform at https://usegalaxy.org in order to check for contamination using the NCBI Foreign Contamination Screen (Astashyn et al. 2024). The workflow used is available here. Ninety-three scaffolds, totalizing 1,591,090 bp, were removed this way, most of them assigned to symbiotic bacteria known to infect *C. lectularius* (*Wolbachia* and Symbiopectobacterium; Hosokawa et al. 2010; Cagatay et al. 2025).

### Sex-chromosome identification

In *C. lectularius*, females are homogametic for the sexual chromosome (XX), while males are heterogametic (XY). In order to identify the X-chromosome in our assembly (because we do not expect the Y, since we sequenced only females), we mapped Illumina reads obtained from the DNA extraction of Harlan strain males (SRA number: SRX498127) or from females (SRA number: SRX15014458), provided by Benoit et al. (2016), onto the chromosome-scale assembly. Because males have only one X, while females have two, half coverage is expected for X chromosome compared to autosomes when mapping reads obtained from males, while no difference is expected when mapping female reads. Mapping was done using bwa-mem2 (v2.2.1) with default parameters and coverage was computed using bedtools (v2.29.1, option genomecov).

### Genetic differentiation and polymorphism assessment

In order to reproduce the differentiation scan done by Haberkorn et al. (2023) on the chromosome-scale assembly, the pool-seq reads (SRA numbers: SRS12605395, SRS12605394) obtained from the two strains differing in resistance [LL and London Field {LF}] were mapped against the newly produced genome assembly using bwa-mem2 (v2.2.1) with default parameters, followed by samtools (v1.20) mpileup. The file produced was then given as input to popoolation2 to call the variants and poolfstat was used to compute the average $F_{\mathrm{ST}}$s using non-overlapping sliding windows of 1,000 Single Nucleotide Polymorphisms (SNPs) (and a minor allele frequency of 0.05). The genetic diversity was also estimated by computing ${\hat{\theta }}_{\pi }$ on a similar sliding window using grenedalf (Czech et al. 2024) and the following parameters : –sam-min-map-qual 50, –pool-sizes pool-sizes.csv, –window-type queue, –window-queue-count 1000, –window-average-policy valid-loci, –filter-sample-min-count 2, –filter-sample-max-count 50, –filter-sample-min-read-depth 4, –filter-sample-max-read-depth 50. Pool sizes were, respectively, 60 and 56 for LF and LL strains. Windows with a mean coverage of >100, most likely corresponding to hidden paralogy due to repeated elements, were considered to be untrustworthy and were discarded. Their inclusion led to a positive (and unexpected) correlation between coverage and ${\hat{\theta }}_{\pi }$ while their exclusion (*n*  = 17 out of 6,340) solved this problem.

### Synteny analysis

The final assembly obtained using the WenganA pipeline and the two haplotypes produced by Miles et al. (2024) were compared in terms of general chromosomal structure using the online software D-genies using default parameters (Cabanettes and Klopp 2018). The synteny plots (dotplots) were obtained using custom R-scripts based on the PAF (Pairwise mApping Format) alignment files produced by D-GENIES.

## Results and discussion

The de novo genome assembly of the “susceptible” strain LL was generated using three sequencing technologies: Illumina short-read sequencing, Oxford Nanopore Technologies long-read sequencing, and Arima Genomics Hi-C sequencing.

### Primary assemblies

Three primary assemblies were obtained, based either on long reads only (corrected by short reads) using flye, or based on both short and long reads using the two flavors of the hybrid assembler Wengan (WenganM or WenganA), and followed by purge_dups in order to remove haplotypic duplications. The total size of the three assemblies was close to the total size of the current reference assembly on NCBI (Clec_2.1), between 494 and 530 Mb (Supplementary Table S1). The BUSCO statistics were overall very good (>97.3% complete BUSCO), although there were slightly more missing genes compared to the reference assembly (between 1.4% and 2.1% compared to 0.3% for the reference). Among the three primary assemblies, the wenganA assembly had overall the best statistics (except for the N50 and number of missing genes), with a total size close to the reference genome (507 Mb).

### Secondary assemblies (after Hi-C scaffolding)

**Fig. 1.**
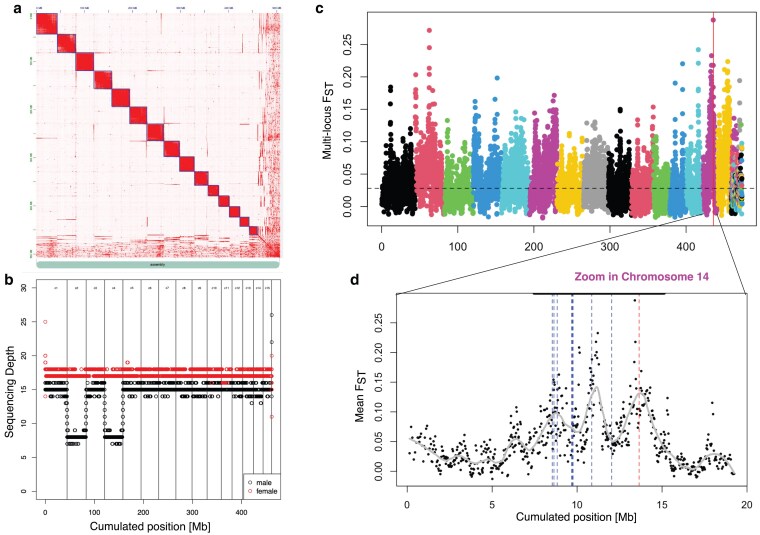
a) Hi-C contact map obtained after WenganA assembly. b) Identification of the X chromosome based on coverage information. The running medians calculated by the runmed R function (with k = 1,001) are plotted for reads obtained from males or females. The different putative chromosomes are separated by vertical bars. c) Whole genome $F_{\mathrm{ST}}$ analysis between the insecticide-susceptible “LL” strain and the insecticide-resistant “LF” strain. The $F_{\mathrm{ST}}$ was computed using a sliding window of 1,000 SNPs (details in the text). The 15 chromosomes are displayed in a decreasing order from left to right and the remaining unplaced scaffolds are plotted on the right, after chromosome 15. The vertical line indicates the position of the *VGSC* gene and the horizontal dashed line indicates the median genome-wide $F_{\mathrm{ST}}$. d) Zoom in C, chromosome 14. The blue lines indicate the positions of 9 out of the 10 putative “resistance genes” depicted in Fig. 3 of Haberkorn et al. (2023) and the red line (around 14 Mb) indicates the position of *VGSC*. The bold line above the plot indicates the genomic region homologous to the four Clec_2.1 scaffolds that compose the “superlocus” as defined in Haberkorn et al. (2023) (now including a region homologous to the scaffold encoding *VGSC* in Clec_2.1). The continuous line was obtained using the loess function in R (span = 0.15) applied on the $F_{\mathrm{ST}}$ values.

Contigs were then scaffolded with 305,559,324 read pairs using YaHS (v1.2). After visual inspection in Juicebox, a minimal number of manipulations was performed for each assembly (Supplementary Fig. S1 and Fig. 1a), resulting in 15 chromosome-sized super-scaffolds for all three assemblies (between 16.4 and 45.2 Mb). As expected, the contiguity of these Hi-C assemblies is much higher compared to the three primary assemblies and to the reference genome, with however slightly fewer complete BUSCO genes detected (between 97.4% and 98.1% compared to 99.6% for the reference assembly Clec_2.1, Table 1). Based on the basic statistics of the assemblies (Table 1), the wenganA assembly was chosen as the best assembly because it obtained good values for all criteria (not necessarily the best but satisfying ones), while it had a much lower number of contigs compared to WenganM and Flye assemblies. The following steps have therefore been performed on this assembly only.

**Table 1.** Statistics of the three assemblies *after* Hi-C scaffolding and comparison with the reference genome (Clec_2.1).

	N contigs	Longest (Mb)	N50 (Mb)	L50	Total (%in chr)	C	F	M	T
*Clec_2.1*	*1,462*	*6.60*	*1.64*	*100*	*510 Mb (100%)*	*2,500*	*3*	*7*	*2,510*
Flye	8,170	46.95	32.77	8	530 Mb (104%)	2,450	27	33	2,510
WenganM	3,125	40.14	32.64	7	494 Mb (96%)	2,447	11	52	2,510
WenganA	2,163	44.27	35.03	7	507 Mb (99%)	2,462	12	36	2,510

C, F, M, and T stand for Complete, Fragmented, Missing, and Total and represent the output from BUSCO pipeline (Hemiptera gene set).

### Sex-Chromosome identification and karyotype

By mapping reads obtained from a Harlan strain male, we identified two super-scaffolds showing the expected 2-fold decrease in sequencing depth (Fig. 1b). These super-scaffolds (2 and 4) most likely correspond to the two fractions of the X chromosome. As expected for X chromosomes, the nucleotide diversity, as measured by $\theta_{\pi }$, was reduced by approximately a factor three-fourth compared to the autosomes for both super-scaffolds [because for 4 copies of an autosome, a population do contain only 3 X {and 1 Y}, see Supplementary Table S3 and Supplementary Fig. S3]. This result further suggests that the super-scaffolds 2 and 4 do correspond to two distinct X chromosomes. Chromosomal fragmentation of the X chromosome has long been observed in the common bed bug *C. lectularius*, as well as in other Cimicidae (Sadílek et al. 2013; Darlington 1939; Slack 1939). Additionally, variation in their number has been noticed, from 2 to 20 putative X chromosomes (Sadílek et al. 2013; Darlington 1939; Slack 1939). In a recent survey (Sadílek et al. 2013), 116 specimens of *C. lectularius* collected over 61 localities spread over Europe revealed a karyotype with 26 autosomes (*n*  = 13), which is consistent with the number of autosomes we found here. In addition, the most common karyotype they found did contain 2 X chromosomes, as observed here.

### Comparison with and among the Harlan strain haplotype assemblies

The Harlan strain was sampled at Fort Dix US Army barracks, NJ, USA, in 1973, and has been maintained since in the lab. By a combination of PacBio reads and Hi-C reads, Miles et al. (2024) produced the assembly of two haplotypes from this strain (hereafter called H1 and H2). The overall structure of the assemblies was consistent with the structure we obtained on the LL strain, with 13 autosomes and 2 X chromosomes. Each chromosome or scaffold of each assembly had a single homologous chromosome in the other assemblies, although they could differ in size, and so their ranking and names (even between H1 and H2, Supplementary Figs. S5 and S6, Supplementary Table S4).

A synteny analysis between all three assemblies (H2 versus H1, H1 versus LL, and H2 versus LL) was conducted (Supplementary Fig. S2). We first compared the two Harlan haplotypes. They were consistent in terms of synteny for five chromosomes (listed above as 1, 2(X1), 6, 7, and 12; Supplementary Fig. S2, third column). In the other chromosome reconstructions, H1 and H2 differ in their architecture. These differences in architecture corresponded mostly to apparent large inversions, intra-chromosomal translocations and inverted translocations. These differences may correspond to true polymorphisms which are known to segregate in natural populations (Wellenreuther and Bernatchez 2018; Gompert et al. 2025). Alternatively, they may correspond to artefacts generated during the assembly process. Remembering that the Harlan strain has been maintained since 1973 as a laboratory colony, such ample polymorphisms in chromosome architecture may sound surprising. Indeed, drift, which necessarily occurred during the more than 50 years of lab rearing, has probably removed most of the diversity within this strain. In addition, haplotype-resolved assemblies from other wild (and thus not inbred) insects (Ye et al. 2023; Agavekar et al. 2025; Gompert et al. 2025) revealed few large-sized structural variation between haplotypes, suggesting that a significant fraction of the differences between H1 and H2 results in fact from artifacts, although we acknowledge that we cannot completely rule out the possibility that some real large-sized structural variants still segregate in the Harlan strain.

We then compared the LL assembly to H1 and H2 (Supplementary Fig. S2, first and second columns). Interestingly, in all five cases where both Harlan haplotypes are consistent (Chr. 1, 2(X1), 6, 7, and 12), the LL assembly was also consistent, giving confidence in the overall structure of these chromosomes. For the other 10 chromosomes, several large differences (>5 Mb) were observed between H1 and H2 assemblies. Still, in most cases, the LL assembly was consistent with either one or the other haplotype reconstruction of the Harlan strain, giving confidence in this haplotype. Finally, for a few chromosomes (number 10, 14) three different architectures were observed depending on the assembly, challenging the identification of the most likely one for these chromosomes.

### Structure of the resistance locus in *C. lectularius*

In order to clarify the gene content and genomic structure of the “resistance” superlocus, we studied the location of the putative “resistance” genes encoded in this locus (the genes highlighted in Fig. 3 of Haberkorn et al. 2023) complemented by the *VGSC* gene (whose genomic location is unclear). To do so, we transferred the annotation of the current reference genome to the LL assemblies and to the two haplotype assemblies of the Harlan strain (Miles et al. 2024) using the pipeline liftoff (see Materials and methods). To the LL, H1, and H2 assemblies, 97%, 93%, and 95% of the exons and 99%, 95%, and 96% of the transcript annotations were transferred respectively, indicating an efficient transfer of the annotations to the new genomes. Using the gff files produced, we then studied the location of the “resistance genes” in the three assemblies (Supplementary Table S2). In all assemblies, most of these genes were located on the same homologous chromosome, which was the second smallest scaffold in the LL assembly (putative chromosome 14) and the smallest one in the Harlan assemblies (putative chromosome 15).

**Fig. 2.**
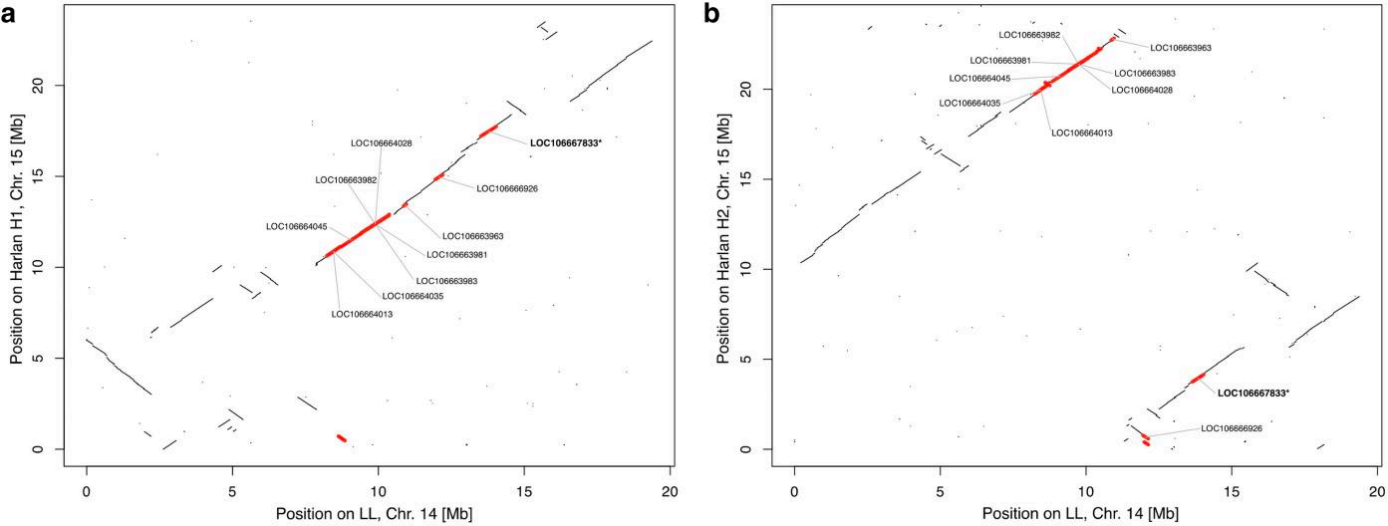
Synteny analysis for chromosome 14 of LL assembly against a) chromosome 15 of Harlan assembly H1 (haplotype 1), b) chromosome 15 of Harlan assembly H2 (haplotype 2). The position of the insecticide resistance genes identified in Fig. 3 of Haberkorn et al. (2023) and described in Supplementary Table S2 is indicated (gene ID from VGSC in bold with a star). Bold (red) lines indicate the synteny blocs overlapping with those genes.

More precisely, the LL assembly indicated that all but one (GST-like) putative insecticide resistance genes were located between positions 7,470,592 and 15,121,489, roughly in the middle of chromosome 14, with VGSC gene located at the extreme end of this locus. Similar genomic organization was observed in the homologous chromosome in Harlan haplotype 1 assembly. However, the assembly of the second Harlan haplotype places VGSC (LOC106667833) and a GST-like (LOC106667926) at the beginning of the chromosome, isolated from all other putative insecticide-resistance genes that cluster at the end of the chromosome in this assembly (Fig. 2). It is unclear which of the three assemblies is the closest to the biological reality. However, all assemblies indicate that most, if not all putative insecticide resistance loci analyzed in Haberkorn et al. (2023), are located on the same chromosome.

### Genetic differentiation at the “superlocus” in LL strain

Using the chromosome-scale assembly obtained from the LL strain, we re-analyzed the pool-seq datasets previously obtained from individuals collected in London (Haberkorn et al. 2023), but showing contrasted resistance phenotypes. The analysis confirmed that the magnitude of the differentiation between the two strains is small (median whole genome $F_{\mathrm{ST}}=0.028$), which makes their comparison valuable to identify adaptive loci. The genome scan revealed that the main differentiation peak between these two strains is located on chromosome 14 (Fig. 1c and Supplementary S4). A closer look at chromosome 14 revealed a 7.65 Mb region with exceptional genetic differentiation (see Supplementary Fig. S7 for a statistical test) encompassing the superlocus identified by Haberkorn et al. (2023), now including the *VGSC* locus (Fig. 1d). In this dataset, the unique known resistance allele in VGSC was L925I (position 13,659,901 on chromosome 14, corresponding to the ATT codon encoding Isoleucine). Its frequency was 0% in the LL strain and 44% in LF strain (Haberkorn et al. 2023). Accordingly, and because we sequenced only the LL strain in the present study, the chromosome-scale assembly contains a CTT codon (encoding Leucine) at this position.

## Conclusion

Using both short and long reads of genomic DNA combined with Hi-C chromosome conformation capture technology, we were able to present here the first annotated chromosome-level assembly of the common bed bug *C. lectularius*. Using this resource, and complementing our analysis with another chromosome-level assembly (Miles et al. 2024), we show that most of the insecticide-resistance genes previously identified (Haberkorn et al. 2023) are located on the same chromosome (number 14 in the LL assembly). More precisely, in the LL assembly, all these genes clustered in a 7.65 Mb superlocus approximately located in the middle of this chromosome. This locus includes GSTs and P450s, and also the VGSC gene. In addition, this locus showed the strongest signal of genetic differentiation between insecticide-susceptible and insecticide-sensitive LL strains. The genomic architecture of the LL assembly was overall consistent in terms of global synteny with either one, the other or both haplotypes from the Harlan strain, except for chromosomes 10 and 14. It is unclear at this stage whether these apparent rearrangements deduced from the comparison of the three haplotypes are biologically relevant or not. Clearly, this warrants additional analysis, especially on chromosome 14 which contains the major locus or loci involved in insecticide resistance. We hope that this resource will be useful to promote future work not only on insecticide resistance evolution, but also on other interesting aspects of the biology of this insect (e.g. ongoing speciation process, as in Booth et al. 2015).

## Supplementary Material

jkaf161_Supplementary_Data

## Data Availability

The assembly, as well as the raw reads, have been deposited in NCBI under Bioproject number PRJNA1207759 (GenBank Assembly GCA_050655605.1). The gff files are available in GSA Figshare: https://doi.org/10.25387/g3.29293874. Supplemental material available at G3 online.
