# Supplementary material for: Chromosome-scale genome assembly of the bed bug Cimex lectularius sheds light on a key insecticide resistance locus
Source: G3 (Bethesda). 2025 Jul 18;15(9):jkaf161. doi: 10.1093/g3journal/jkaf161 (PMC12405873; doi:10.1093/g3journal/jkaf161)
Supplement: jkaf161_Supplementary_Data [file jkaf161_supplementary_data.pdf]

Supplemental Material for Haberkorn, Varaldi *et al.* 2025

	N contigs	longest	N50	L50	total (Mb)	C	F	M	T
Clec_2.1	*1462*	*6600950*	*1637644*	*100*	*510 (100%)*	*2500*	*3*	*7*	*2510*
**Flye**	11845	941499	156431	1000	530 (104%)	2446	30	34	2510
**WenganM**	7848	976376	116935	1244	494 (96%)	2442	15	53	2510
**WenganA**	6383	1069240	149662	976	507 (99%)	2458	14	38	2510

Table S1: Statistics of the three assemblies *before* Hi-C scaffolding and comparison with the reference genome (Clec_2.1). C, F, M and T stands for Complete, Fragmented, Missing and Total and represent the output from BUSCO pipeline (Hemiptera gene set).

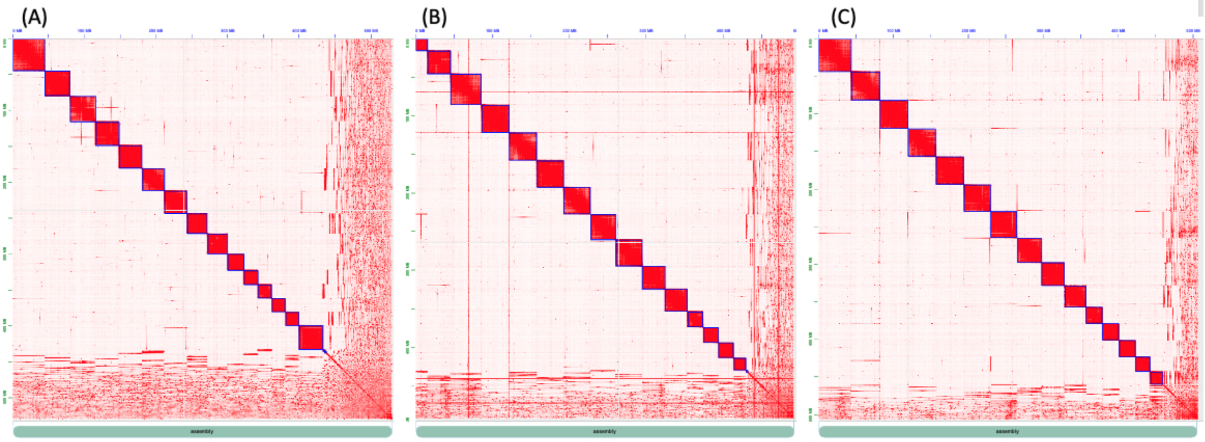

Figure S1: Hi-C contact map obtained using the three primary assemblies: (A) flye, (B) WenganM, (C) WenganA.

GeneID	Protein product	Flye	WenganM	WenganA*	H1	H2
LOC106663963	GST-like	scaffold_13	scaffold_14	scaffold_14	chr_15	chr_15
LOC106663981	CYP6B1-like	scaffold_13	NA	scaffold_14	chr_15	chr_15
LOC106663982	CYP6B1-like	scaffold_1402	scaffold_14	scaffold_14	chr_15	chr_15
LOC106663983	CYP6B5	scaffold_13	scaffold_14	scaffold_14	chr_15	chr_15
LOC106664013	choline-O-acetyltransferase	scaffold_13	scaffold_14	scaffold_14	chr_15	chr_15
LOC106664023	GST-like	scaffold_13	scaffold_847	scaffold_845	chr_15	chr_15
LOC106664028	SMPDL3a	scaffold_13	scaffold_14	scaffold_14	chr_15	chr_15
LOC106664035	vesicular acetylcholine transporter	scaffold_2810	scaffold_14	scaffold_14	chr_15	chr_15
LOC106664045	high-affinity choline transporter 1	scaffold_13	scaffold_14	scaffold_14	chr_15	chr_15
LOC106666926	GST-like	scaffold_13	scaffold_14	scaffold_14	chr_15	chr_15
LOC106667833	VGSC	scaffold_28	scaffold_14	scaffold_14	chr_15	chr_15

Table S2: Location of 11 "resistance genes" (with their protein product) in the three London Lab assemblies (this study) and the two Harlan haplotypes assemblies (H1, H2, after [5]) obtained after Hi-C scaffolding. The scaffold numbers do not necessarily reflect their size. The "best" assembly among London Lab assemblies was identified as WenganA* based on summary statistics (see text for details). Importantly, the *VGSC* gene (LOC106667833) was consistently found on the same scaffold together with most of the other insecticide-resistance genes in wenganM and wenganA assemblies. For the Flye assembly, an additional visual inspection of the contact map clearly revealed that the scaffold encoding *VGSC* in this assembly (scaffold_28) was uniquely connected to scaffold_13 that contain most of the other putative insecticide-resistance genes.

This work is shared under a CC BY-SA 4.0 license unless otherwise noted

Strain	Autosomes	Expected (on X)	X1	X2	X
LL	0.005500	0.004125	0.003431	0.003530	0.003480
LF	0.005578	0.004183	0.003795	0.003480	0.003637

Table S3: Average nucleotidic diversity ($\hat{\theta}_\pi$) in autosomes and in the putative X chromosomes. $\hat{\theta}_\pi$ were computed using 1000 SNPs windows using the 'Valid loci' option of grenedalf, and the median of $\hat{\theta}_\pi$ was computed for each chromosome separately. The average nucleotidic diversity is given for individual X chromosome separately (X1 and X2 corresponding respectively to scaffold_2 and scaffold_4) and for the whole X chromosome (that includes both scaffold_2 and scaffold_4). The expected value for X diversity is the 3/4 ratio compared to autosomes.

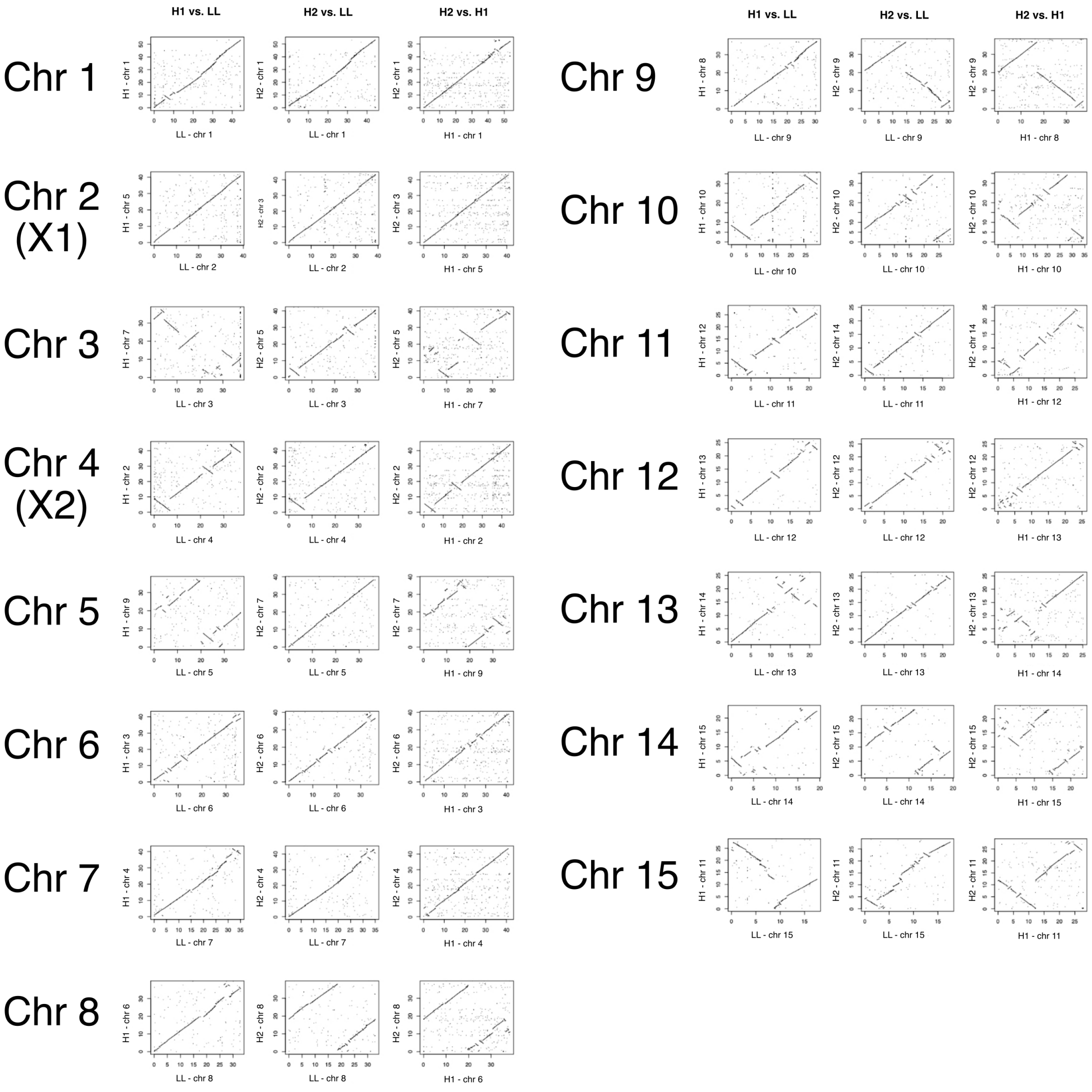

Figure S2: Synteny analysis between London Lab (LL), Harlan haplotype 1 (H1) and Harlan haplotype 2 (H2). The first column represents the synteny between H1 and LL, the second column the synteny between H2 and LL, and the third one the synteny between H1 and H2. Each row corresponds to a putative chromosome, named according to the LL assembly. Size in Mb.

This work is shared under a CC BY-SA 4.0 license unless otherwise noted

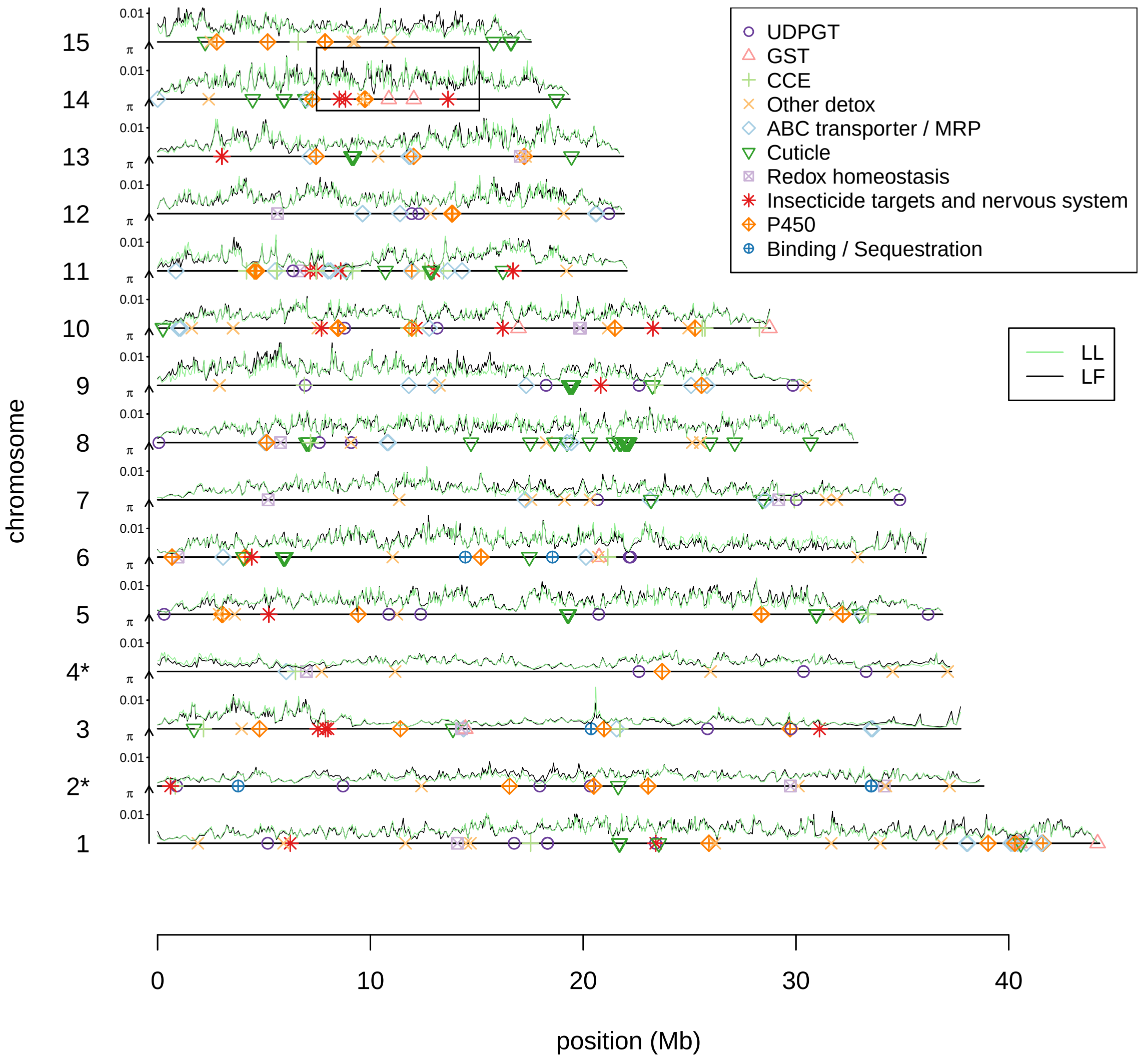

Figure S3: Overview of the nucleotidic diversity, as measured by θ_π for the insecticide-susceptible "London Lab" strain (LL) and the insecticide-resistant "London Field" strain (LF). The different genes classified as potentially involved in insecticide-resistance are depicted as coloured crosses (the classification of these genes in functional categories is from Haberkorn *et al.* 2023 [4]). A box surrounds the "superlocus" in chromosome 14, and the putative X chromosomes are indicated by a star (Chr. 2 and 4).

This work is shared under a CC BY-SA 4.0 license unless otherwise noted

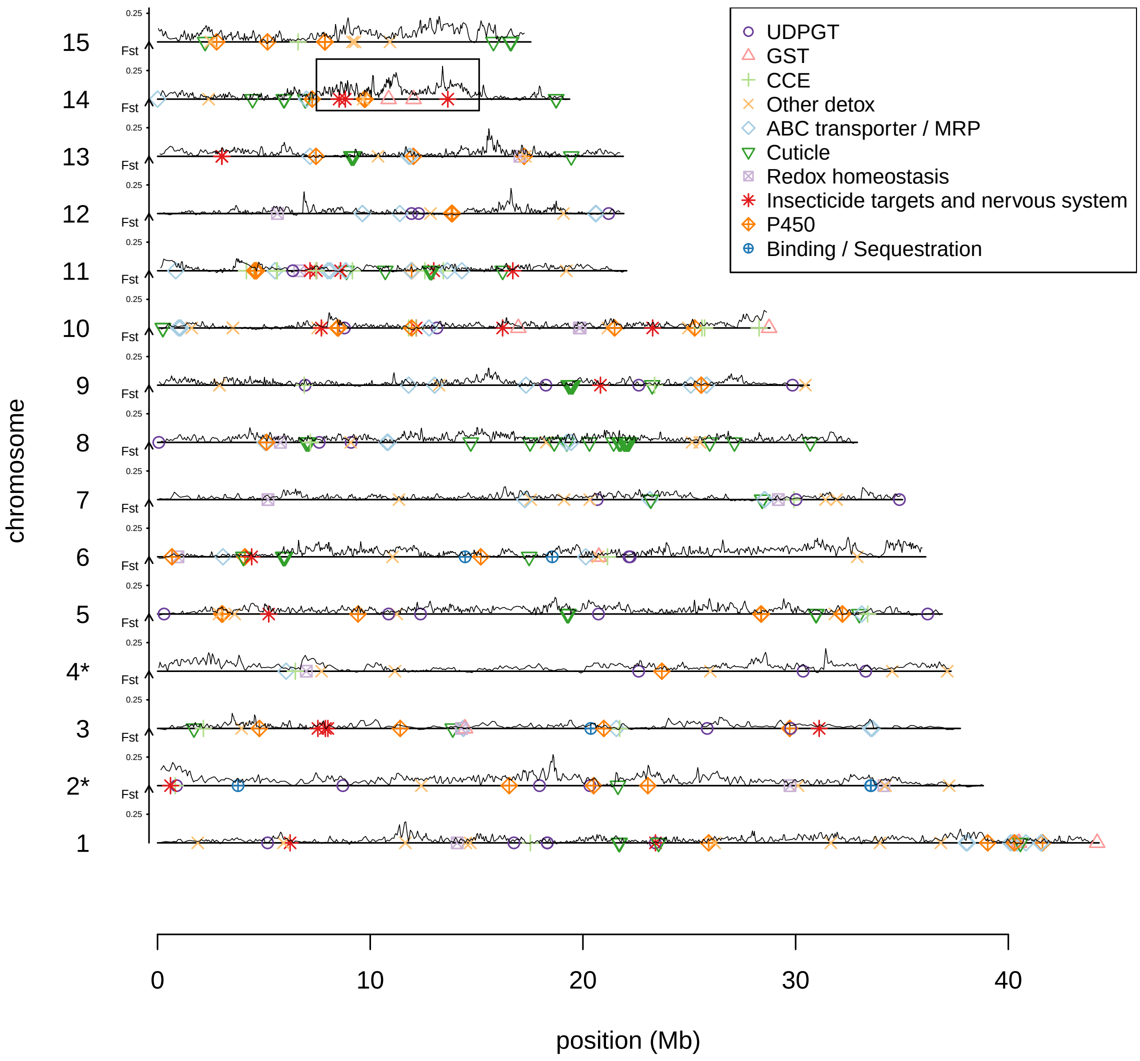

Figure S4: Overview of the genetic differentiation, as measured by the F_{ST}, between the insecticide-susceptible "London Lab" strain (LL) and the insecticide-resistant "London Field" strain (LF). The different genes classified as potentially involved in insecticide-resistance are depicted as coloured crosses (the classification of these genes in functional categories is from Haberkorn *et al.* 2023 [4]). A box surrounds the "superlocus" in chromosome 14, and the putative X chromosomes are indicated by a star (Chr. 2 and 4).

This work is shared under a CC BY-SA 4.0 license unless otherwise noted

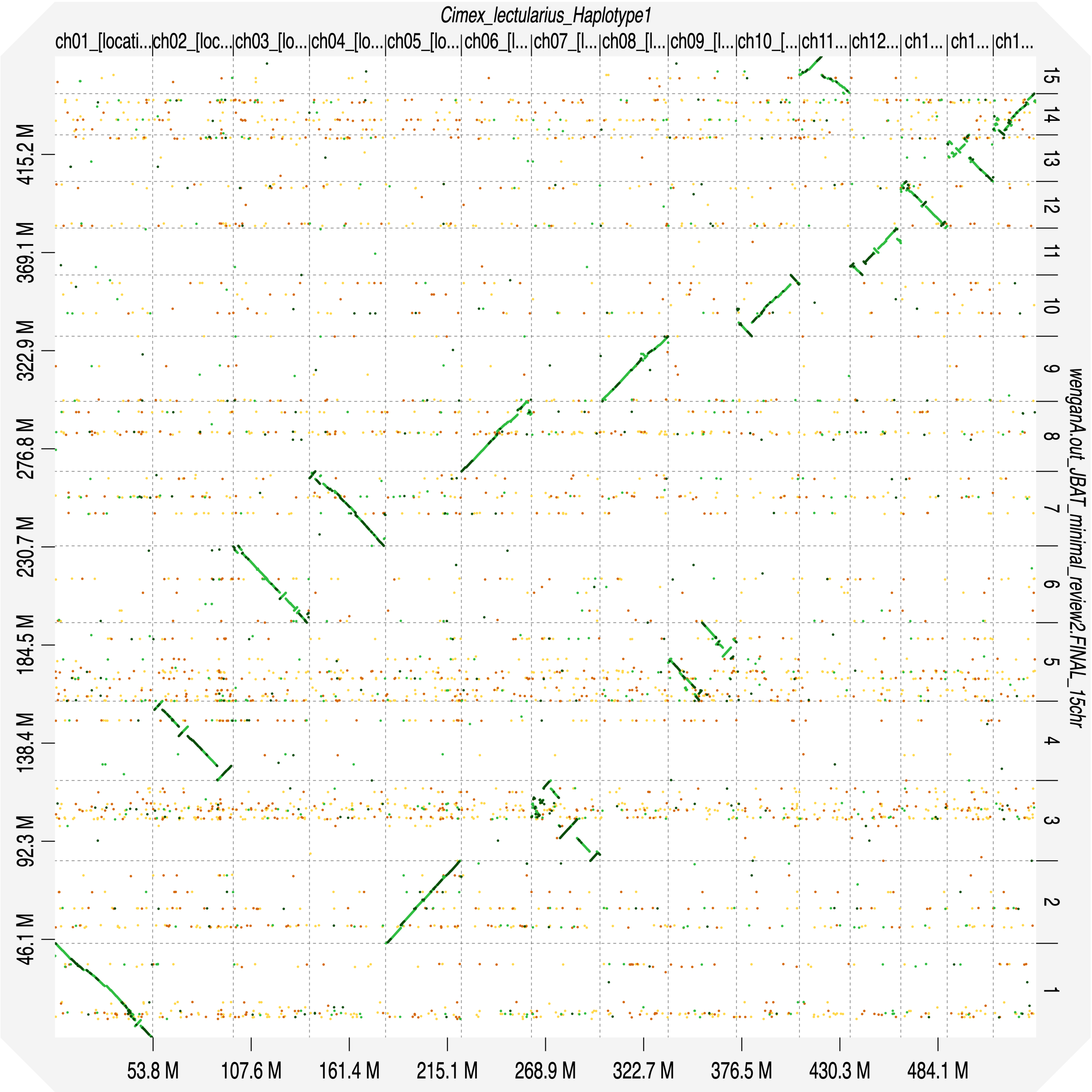

Figure S5: Comparison between Haplotype 1 (Harlan) assembly and the LL (London Lab) assembly. The plot was obtained using the online version of D-Genies [27]

This work is shared under a CC BY-SA 4.0 license unless otherwise noted

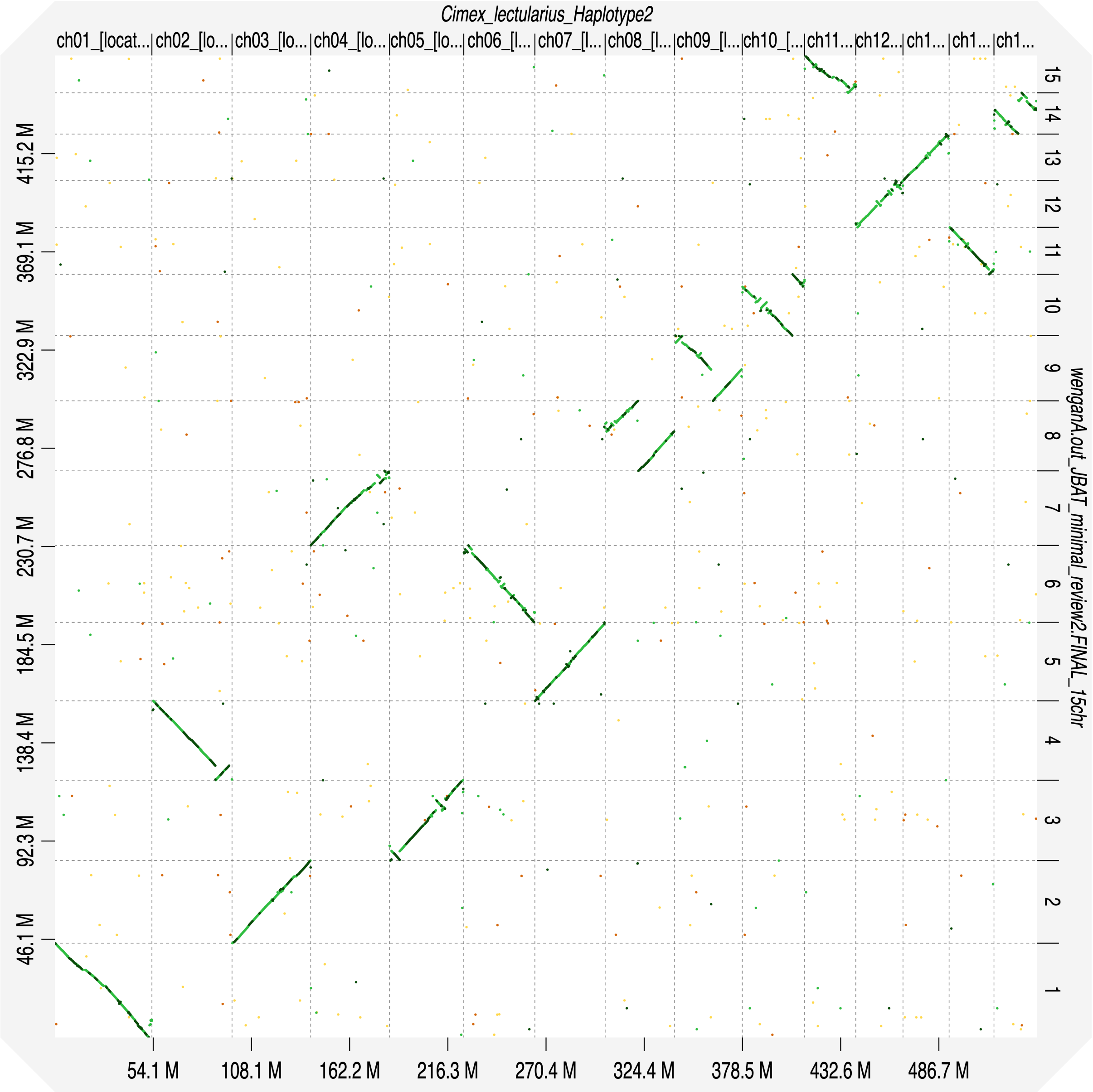

Figure S6: Comparison between Haplotype 2 (Harlan) assembly and the LL (London Lab) assembly. The plot was obtained using the online version of D-Genies [27]

This work is shared under a CC BY-SA 4.0 license unless otherwise noted

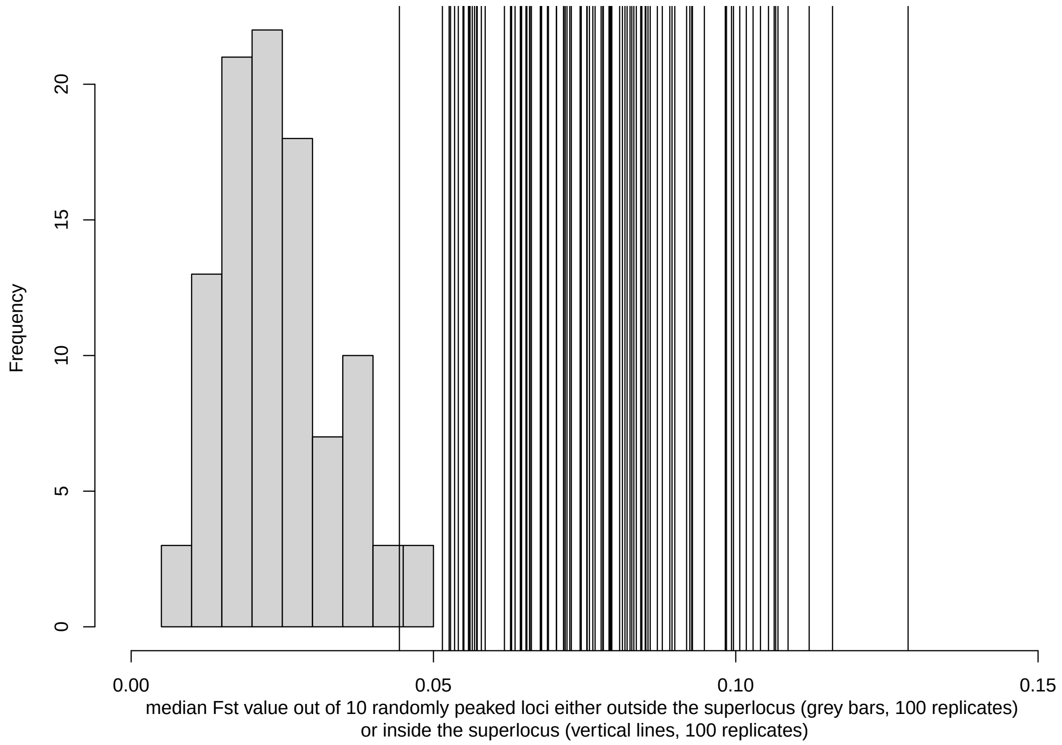

Figure S7: The "superlocus" (between positions 7,47 Mb and 15,12 Mb on Chr. 14) is exceptionally differentiated. Ten randomly peaked loci were sampled either outside this region (within Chr. 14) or inside this region (without replacement) and the median F_{ST} values were calculated. This procedure was repeated 100 times. The grey bars show the distribution of the median for loci outside the region while the vertical bars show the values obtained inside the locus.

This work is shared under a CC BY-SA 4.0 license unless otherwise noted

LL	H1	H2
1	1	1
2(X1)	5	3
3	7	5
4(X2)	2	2
5	9	7
6	3	6
7	4	4
8	6	8
9	8	9
10	10	10
11	12	14
12	13	12
13	14	13
14	15	15
15	11	11

Table S4: Homologous chromosome numbering among the three assemblies. LL : London Lab, H1: Harlan haplotype 1, H2: Harlan haplotype 2.

This work is shared under a CC BY-SA 4.0 license unless otherwise noted
